# Harmonizing neuropathic pain research: outcomes of the London consensus meeting on peripheral tissue studies

**DOI:** 10.1097/j.pain.0000000000003445

**Published:** 2024-10-16

**Authors:** Sara Villa, Eske K. Aasvang, Nadine Attal, Ralf Baron, Emmanuel Bourinet, Margarita Calvo, Nanna B. Finnerup, Eleonora Galosi, James R.F. Hockley, Pall Karlsson, Harriet Kemp, Jannis Körner, Ekaterina Kutafina, Angelika Lampert, Margarita Mürk, Zahra Nochi, Theodore J. Price, Andrew S.C. Rice, Claudia Sommer, Pille Taba, Andreas C. Themistocleous, Rolf-Detlef Treede, Andrea Truini, Nurcan Üçeyler, David L. Bennett, Annina B. Schmid, Franziska Denk

**Affiliations:** a Wolfson Sensory, Pain and Regeneration Centre (SPaRC), King's College London, United Kingdom; b Anesthesiological Department, Center for Cancer and Organ Dysfunction, Rigshospitalet, Copenhagen University, Copenhagen, Denmark; c INSERM U987, APHP, UVSQ Paris SACLAY University, Hôpital Ambroise Paré, Boulogne-Billancourt, France; d Division of Neurological Pain Research and Therapy, Department of Neurology, Christian-Albrechts-Universität Kiel, Kiel, Germany; e Laboratories of Excellence, Ion Channel Science and Therapeutics, Institut de Génomique Fonctionnelle, Montpellier, France; f CNRS UMR5203, Montpellier, France; g INSERM, U661, Montpellier, France; h Université de Montpellier, Montpellier, France; i Biological Sciences Faculty and Faculty of Medicine, Pontificia Universidad Católica de Chile Santiago, CL, United States,; j Department of Clinical Medicine, Danish Pain Research Center, Aarhus University, Aarhus, Denmark; k Department of Human Neuroscience, Sapienza University, Rome, Italy; l GSK, Stevenage, United Kingdom; m Pain Research Group, Department of Surgery and Cancer, Imperial College London, London, United Kingdom; n Institute of Neurophysiology, Uniklinik RWTH Aachen University, Aachen, Germany; o Department of Anesthesiology, Uniklinik RWTH Aachen University; p Intensive and Intermediate Care, Uniklinik RWTH Aachen University, Aachen, Germany; q Institute for Biomedical Informatics, Faculty of Medicine, University Hospital Cologne, University of Cologne, Cologne, Germany; r Scientific Center for Neuropathic Pain Research Aachen, SCN^AACHEN^, Uniklinik RWTH Aachen University, Aachen, Germany; s Pathology Department, Tartu University Hospital, Tartu, Estonia; t Department of Neuroscience and Center for Advanced Pain Studies, University of Texas at Dallas, Richardson, TX, United States; u Department of Neurology, University Hospital Würzburg, Würzburg, Germany; v Department of Neurology and Neurosurgery, University of Tartu, Tartu, Estonia; w Nuffield Department of Clinical Neurosciences, University of Oxford, Oxford, United Kingdom; x Department of Psychiatry and Psychotherapy, Central Institute for Mental Health, Medical Faculty Mannheim, Heidelberg University, Mannheim, Germany; y Department of Neurology, University Hospital Würzburg, Würzburg, Germany

**Keywords:** Neuropathic pain, Peripheral nerve, Dorsal root ganglia, Protocol harmonization, Inflammation, Neuro-immune

## Abstract

Neuropathic pain remains difficult to treat, with drug development hampered by an incomplete understanding of the pathogenesis of the condition, as well as a lack of biomarkers. The problem is compounded by the scarcity of relevant human peripheral tissues, including skin, nerves, and dorsal root ganglia. Efforts to obtain such samples are accelerating, increasing the need for standardisation across laboratories. In this white paper, we report on a consensus meeting attended by neuropathic pain experts, designed to accelerate protocol alignment and harmonization of studies involving relevant peripheral tissues. The meeting was held in London in March 2024 and attended by 28 networking partners, including industry and patient representatives. We achieved consensus on minimal recommended phenotyping, harmonised wet laboratory protocols, statistical design, reporting, and data sharing. Here, we also share a variety of relevant standard operating procedures as supplementary protocols. We envision that our recommendations will help unify human tissue research in the field and accelerate our understanding of how abnormal interactions between sensory neurons and their local peripheral environment contribute towards neuropathic pain.

## Abstract 2

Supplemental Digital Content is Available in the Text.

## 1. Introduction

Of all chronic pain conditions, neuropathic pain is particularly devastating. Its impact on health-related quality of life is substantial, with traditional analgesics unable to offer adequate relief and causing significant side effects in a substantial proportion of individuals. We therefore urgently need novel and collaborative research approaches to help the millions of people living with neuropathic pain; it is estimated to be as many as 9% of the population in Europe alone,^2,4^ that is 56 million adults in absolute numbers—almost equivalent to the entire population of Italy.

One very significant problem that hampers scientific progress in this area is the scarcity of data derived from human tissue. This very much limits our ability to discover drug targets and assess the translational value of cell and animal models. A particularly important blind spot is the precise nature of the local tissue environment that nerve fibres and their cell bodies in the dorsal root ganglion (DRG) are situated in. The complex neuro-immune-stromal cell interactions that take place in these peripheral surroundings are thought to be key determinants of nociceptive neuron activation and sensitization.

Yet, because of the difficulty in obtaining relevant tissues, these interactions have barely been characterized in human neuropathic pain conditions, creating significant knowledge gaps. There is a reliance on animal models, mostly of nerve injury and to a lesser extent neuropathy with defined aetiology. However, there are significant species differences in sensory neuron gene expression,^27,57^ as well as the neuronal circuitry^63^ and inflammatory processes relevant to chronic pain conditions.^6,23^ Moreover, human neuropathies are often slowly evolving while animal models are usually studied acutely, with some notable exceptions.^26,34^ The latter indicate the presence of long-lasting inflammation in neuropathic pain, as do the few limited studies that have been conducted in human skin,^24,56^ DRG,^16,25,40,44^ and nerve.^50^ Yet, the details of peripheral immune and mesenchymal cell (dys)function remain to be fully elucidated.

The best way to remedy this and increase our understanding of local inflammation in neuropathic pain is to join forces and generate larger collections of pain-relevant human tissues and samples with matched clinical data as well as relevant control tissue.^47^ Here, we report on a consensus meeting funded by the European Union's ERA-NET Neuron scheme, designed to accelerate such efforts by making practical recommendations for better protocol alignment and harmonization across research groups. The meeting was attended by a group of 28 experts in the field of neuropathic pain research, including industry and patient partners, who came together for a 2-day face-to-face event in London on March 18 and 19, 2024.

## 2. Initial survey and topic selection

In anticipation of the meeting, an online survey was conducted to help choose the focus of our discussions. It was sent out to 33 network partners in June 2023 and received a 57% response rate. The group included 2 clinicians, 14 clinician scientists of varying specialities, like neurology, anaesthesiology, and physiotherapy, 12 basic scientists, 4 industry representatives, and 1 patient expert. A pdf of the full survey results can be viewed in Supplementary File 1 (http://links.lww.com/PAIN/C157). Briefly, 74% of survey respondents indicated that their resources of human tissues or samples were obtained as part of their own work, whereas 26% used biobanks or collaborators as a source. This was expected because the majority of our network was deliberately selected to include individuals who have access to human tissue for neuropathic pain research. The most commonly available tissue types were skin biopsies (79% of respondents) and whole blood and serum (68%), followed by nerves (42%) and DRG (32%). Detailed phenotyping data are being collected from participants providing these samples, with 68% of respondents obtaining data not just through pain questionnaires and clinical examination, but also through quantitative sensory testing (QST) and assessments of psychological factors and health-related quality of life. Fifty-eight percent of respondents reported assessing nerve conduction and 26% the use of microneurography, which allows the study of nociceptive C-fibres. When asked about the biggest obstacles to harmonization across research groups, respondents noted regulatory constraints, lack of standardized tissue processing and phenotyping protocols, and lack of funds among their primary concerns.

Based on the survey data and initial discussions at the face-to-face meeting, 3 topics were chosen for in-depth consensus seeking: minimal phenotyping, protocol alignment, and reporting and data sharing. We also decided to focus on the tissues that were most readily available within the group, specifically skin biopsy, blood, nerve, and DRG samples. The consensus meeting itself was attended by 28 network participants, including 1 patient expert (S.V.) and 2 industry partners. Each topic was briefly introduced and then discussed at roundtables with 7 people of mixed expertise. The conversations at each table were then presented and further debated in an open forum to reach consensus.

## 3. Minimal phenotyping

Phenotyping standards were discussed at length, and consensus outcomes are summarised in Table 1. The group agreed that neuropathic pain should be categorised using the updated International Association for the Study of Pain Neuropathic Pain Special Interest Group (NeuPSIG) grading criteria^20^ and assessed in accordance with the latest European Academy of Neurology, European Pain Federation (EFIC), and NeuPSIG guidelines.^60^ Ideally, a study on neuropathic pain would only include individuals that are graded as having a definite neuropathic pain diagnosis (and matched controls if the design requires). The label of “definite” necessitates pain and sensory signs in an anatomically plausible distribution and a diagnostic test^60^ to confirm a lesion or disease of the somatosensory nervous system.^20^ However, the group acknowledged that a definite diagnosis may not always be possible because of time or financial constraints or because of the absence of a valid diagnostic test, eg, in the case of radicular pain. In those instances, we would recommend the inclusion of “probable neuropathic pain,” ie, eliminating the need for a definitive diagnostic test, or at the very least, the correct grading of each participant according to the established criteria. Thus, at a minimum, a study should include information on the neuropathic pain grading determined for each participant, to enable later division of any study data into “possible,” “probable,” or “definite” neuropathic pain categories.

**Table 1:** Consensus recommendations for minimal, extended, and postmortem phenotyping.

Domain	Minimal phenotype	Extended phenotype	Postmortem phenotype
Demographics	Age, biological sex, height, weight	Age, sex, gender, height, weight	Age, sex, gender, height, weight
General health information		Smoking, alcohol consumption, recreational drug use (especially nitrous oxide), presence of co-morbidities	Presence of co-morbidities
Medication history	Past or present neurotoxic medications or treatments, current analgesics, and strong immunomodulators	Past or present neurotoxic medications, current analgesics and strong immunomodulators	Past or present neurotoxic medications, current analgesics, and strong immunomodulators
Presence of neuropathic pain	Neuropathic pain grading tool	Neuropathic pain grading tool	Presence and type of pain if available
Pain severity, pain quality	Numerical pain rating scale specific to the site of neuropathy, eg, using a body map	Numerical pain rating scale specific to the site of neuropathy, Neuropathic Pain Symptom Inventory	
Pain interference		Brief Pain Inventory, disease-specific measure(s) if available	
Activities of daily living		Brief pain Inventory, disease-specific measure(s) if available	
Quality of life		EQ-5D	
Diagnosis		ICD-11, plus additional subdiagnoses as needed	
Clinical examination	If conducted as part of neuropathic pain grading	Neurological examination including tendon reflexes, muscle strength, sensation (light touch, vibration, pin prick, thermal), if available sympathetic testing	
Somatosensory profiling		Quantitative sensory testing, including bedside	

EQ-5D, EuroQol-5D.

Once neuropathic pain diagnosis is established, it was discussed that it would be valuable to start with domains that capture different aspects of the neuropathic pain experience. Core outcome sets for chronic pain have recently been agreed upon by the INTEGRATE-pain Consortium using a Delphi process.^5^ There was broad agreement that these would also be suitable for the purposes of studying local tissue dysregulation in neuropathic pain. For chronic pain, these must include a measure of pain intensity and interference, as well as assessment of how pain affects activities of daily living and quality of life. Beyond these broader recommendations, our group also converged on more specific suggestions regarding phenotyping in studies that involve local tissue. We split discussions into those studies that would use donated samples from living individuals vs postmortem designs.

In the case of the former, it was discussed that, for statistical purposes, many tissue studies probably would not be large enough to be powered for more than one outcome variable, hence deviation from core outcome sets could be justified in these specific cases. We discussed the absolute minimum data required for such studies in addition to NeupSIG grading, and agreed on age, biological sex, height, weight, a measure of pain severity to serve as the primary outcome, and some information on pain medications. Height and weight were considered more flexible measures than body mass index and therefore preferable. All agreed that it was vital that any pain assessment be specific to the site of neuropathy, for instance, in the context of typical diabetic neuropathy, enquire about pain in the feet. This is important because patients often experience other types of pain, eg, musculoskeletal. Similarly, it should be recorded how pain assessment related to the site of the tissue that was being harvested—perhaps with the help of a body map. There was much debate regarding the actual minimal pain severity metric to be used. Suggestions varied and included the total intensity score of the Neuropathic Pain Symptom Inventory or an average numerical rating score of pain intensity over the past 24 hours, with the latter preferred by most of the group. In conditions with intermittent pain, this might have to be modified, eg, to ask about the intensity of the last attack or the frequency of attacks. Finally, regarding medications, as an absolute minimum, there was agreement that information was needed on past or present neurotoxic medications or treatments (eg, chemotherapy or radiotherapy), as well as current analgesics and strong immunomodulators like biologic therapies. Obviously, precise details on what might be most appropriate would depend on the neuropathic pain condition and tissue site under study. We recommend adapting a short record sheet that captures the minimal phenotyping information we just discussed, alongside a few additional example parameters (Supplementary File 2, http://links.lww.com/PAIN/C157).

If a study allows for broader phenotyping beyond the minimum, there were several additional suggestions. In terms of demographics, it was proposed to record gender, smoking, alcohol consumption, recreational drug use, and the presence of other co-morbid chronic diseases. In terms of questionnaires, there were several suggestions that would cover the INTEGRATE core outcome sets, for example, combining the Brief Pain Inventory, Neuropathic Pain Symptom Inventory, and the EuroQol-5D assessment of health-related quality of life. This was estimated to take about 20 minutes per patient. Clinically, use of the new ICD-11 classification system was proposed as a good option^39^ because it includes extension codes for pain intensity, distress, disability, and psychosocial factors. A neurological examination should also be conducted, including a detailed assessment of somatosensory function. This was suggested to include tendon reflexes, muscle strength, as well as thermal, vibration, light touch, and pin prick testing to assess sensory deficits, hypoalgesia/hyperalgesia, and allodynia. Autonomic symptoms were also mentioned, as they frequently accompany neuropathic pain syndromes.^14^ Finally, the use of QST was proposed, especially for studies investigating small nerve fibre neuropathy (in which standard large-fibre nerve conduction is often normal). Both bedside QST^3,46,49,64^ or the complete German Research Network on Neuropathic Pain (DFNS) protocol^48^ are suitable, depending on the research question.

In contrast to tissues and samples from living individuals, postmortem tissue collection has its own set of challenges with regards to phenotyping. Demographics are likely available and should be collected, especially age, biological sex and gender, height, and weight. Similarly, detailed clinical records may be accessible, as was the case for our 2 expert panel members who regularly obtain postmortem human DRG tissue (T.J.P. and E.B.). However, although these give information on co-morbidities and past medications, gaining more insight into specifics of neuropathic pain conditions can depend on the availability of family history and medical records and may not always be available. The option of deriving information from next of kin was discussed but depends heavily on the way organ procurement works in different countries; and it was agreed that family privacy is paramount. One interesting suggestion was to take distal (10 cm above the lateral malleolus) and proximal (upper lateral aspect of the thigh, 20 cm below the anterior iliac spine) skin biopsies that, by comparing to reference data,^11,32,43^ might allow for postmortem confirmation of a reduction in intraepidermal nerve fibre density (IENFD), consistent with possible neuropathy. However, it would remain unknown in many cases whether this was painful or not and would also only provide information on potential neuropathies affecting the lower limb in a length-dependent manner. Work in this area could benefit from a standardized questionnaire that could be included in information that is collected from next of kin during the organ procurement process.

## 4. Protocol alignment

The discussion on sample processing was divided into tissue type (skin, nerve and DRG, blood) and downstream technique. In all instances, human biological samples must be sourced ethically in line with the Declaration of Helsinki, with their research use approved by an appropriate ethical review committee and in accord with the terms of informed consent. Regarding technical protocols, it was acknowledged that advances continue to be made, which means that detailed methods can quickly become obsolete. Here, we therefore share the protocols we currently use or recommend using Zenodo within a newly set up ERA-NET EndPain Community group.^18^ Zenodo is a general-purpose open repository developed under the European OpenAIRE program and operated by CERN. Protocols are version-controlled and citable, with the system also allowing future groups to tag their neuropathic pain relevant human tissue protocols to the ERA-NET EndPain Community.

### 4.1. Skin

For immunostaining of skin biopsy, the group strongly agreed that we should adhere to existing guidelines^33^ because these were used to assemble current reference datasets. However, there was also agreement that additional detail around these guidelines could be helpful. We are therefore providing a range of protocols currently used by expert groups in the field.^21,28,29,58^ They contain more information on how to take the biopsy, including contraindications and expected adverse events, to help with ethical approvals. Regarding ethics, we suggest that patients who undergo skin biopsies for diagnostic purposes are routinely consented for any left-over biopsy tissue to be used for future research.

We also discussed fixatives, and the experience of our attendees was in keeping with the 2010 guidelines: Zamboni^21,29^ and periodate-lysine-paraformaldehyde fixatives^28^ were most widely used, although it was also agreed that tissue fixed with 4% paraformaldehyde can permit visualisation of skin IENFD to a reasonable extent, although with more fragmented morphology (see 58 for example SOP). The consensus was that preference for the former 2 fixation agents was now largely by default: most large-scale studies, including IENFD reference data, have been generated with their help, while side-by-side comparisons with other fixation agents are lacking. It was noted that Zamboni might be less favoured by health and safety officials because of it containing picric acid, which presents an explosion hazard when dried. Finally, it has been the experience of workshop attendees that some immune and stromal cell antibodies commonly used in the immunology field do not work as well in periodate-lysine-paraformaldehyde. We therefore anticipate that new antibody panels will need to be generated to allow for the study of neuro-immune-stromal cell interactions in an optimal fashion.

On a more conceptual level, the absence of normative IENFD data for non-White populations and individuals younger than 18 and older than 80 years was noted as a significant shortcoming, as well as the lack of healthy control biopsies for diverse body sites. Studies concerning such cohorts should be supported, as well as the development of automated fibre counting algorithms. In all cases, we would like to highlight that although both immunohistochemistry and immunofluorescence are equally suitable for assessing IENFD, the techniques do yield different absolute counts and therefore have to be compared with technique-matched normative datasets. We recommend Provitera et al.^43^ for indirect immunofluorescence and Lauria et al.^32^ for bright field immunohistochemistry, as well as using 50-μm sections to match the data that are available. There was also a suggestion that the field might want to start consider using z-scores, as is done for QST, to facilitate comparisons across site, sex, and age.

### 4.2. Nerve and dorsal root ganglion

Nerves can be obtained as surplus tissue after surgery,^51^ in the form of diagnostic biopsies or postmortem. For diagnostic biopsies, a number of guidelines have been published over the years.^55,62^ For DRG,^53^ we recommend that anatomical levels are recorded where possible because there may be functional and structural differences between lumbar, thoracic, and cervical DRG. With long pieces of nerve, it can be useful to have a permanent indication of proximal vs distal orientation. For pipelines not dependent on living neurons (eg, for electrophysiological studies), most groups fresh-freeze their tissue in either liquid nitrogen or pulverized dry ice^1^ and later postfix in 4% paraformaldehyde or 10% formalin for immunostaining. Dorsal root ganglion immunostaining is nontrivial because of the high levels of autofluorescence emitted from lipofuscin, which is contained in almost all adult DRG neurons. Some teams therefore currently rely on chromogenic RNA-scope to avoid having to use fluorescence imaging, whereas others use an open-channel method for subtracting background from lipofuscin in fluorescent RNA-scope imaging.^44,57^ Another, as yet unresolved issue is quantification, which can be very difficult and time-consuming in human DRG.

To maximise protocol flexibility, it may often be advisable to store precious human tissues in several “formats,” eg, cutting nerves in half or processing each of the 2 DRGs at each level differently. For example, one piece could be kept to allow for evaluation of cells “in situ,” eg, using RNAscope, immunostaining, or spatial transcriptomics, whereas another piece could be processed to maximise downstream molecular and cellular analysis of nonneuronal cells. For nonneuronal cells, an option used by many groups is freezing tissue in Cryostor CS10 or similar freezing reagents that permit recovery of most cell-types and their transcriptomes, for fluorescence-activated cell sorting or single-cell RNA sequencing.^15^

### 4.3. Blood

Our group felt that systemic biomarkers derived from serum or peripheral blood mononuclear cells (PBMCs) are more likely to suffer from poor sensitivity, especially in the case of focal neuropathies, where abnormal neuro-immune communications may be confined to local resident tissues. Regardless, concomitant collection of whole blood, serum, or PBMCs was still advised where possible and has proven utility in polyneuropathies that accompany systemic disorders like diabetes.^35^ It might allow merging of cohorts into large-scale studies with better power to detect relatively small changes, as well as the use of patient-matched PBMCs for mechanistic in vitro studies (eg, using induced pluripotent stem cell-derived sensory neurons). Of course, relevant consent for sample sharing would need to be sought—ideally at the point of sample collection.

## 5. Statistical design, reporting, and data sharing

To maximise the information that can be garnered from precious human tissue generously donated, we would strongly recommend preregistering statistical design and analysis plans and protocols, even outside the context of a formal clinical trial.^54^ This can be done formally, eg, as a published study protocol, or informally, on time-stamped repositories like the Open Science Framework. Preregistration does not preclude any exploratory analyses from taking place, but simply ensures that a priori hypotheses can be unambiguously identified, greatly strengthening the statistical inferences that can be drawn.^41^

There was also broad consensus among our participants that adherence to reporting standards was paramount, not least because it might allow meta-analyses to proceed in cases where sharing of raw data is hampered by ethical and data protection constraints. We advise the use of established guidelines where possible, like CONSORT for randomised clinical trials,^52^ STROBE for observational studies,^61^ and STARD^9^ and TRIPOD^10^ for diagnostic and prognostic studies, respectively. Several guidelines relevant to tissues implicated in neuropathic pain also already exist, see, eg, the repository provided by the Equator Network web site.^17^ These include reporting on cellular and molecular data within a clinical trial (SPRIT-Path extension^30^), reporting on human biospecimens,^37^ and the EULAR minimal reporting guidelines for synovial tissue research.^38^ None of them are specific to the tissues we focused on in our workshop. We therefore also made some additional recommendations for reporting on tissues collected for studies of neuropathic pain.

It was considered vital to report the precise site of tissue collection, for example, where a skin or nerve biopsy was taken in relation to the nerve injury or which anatomical level a DRG derives from, and how this relates to the clinical data that were collected. Duration and condition of storage should be disclosed, as well as postmortem interval, if applicable. Quality control measures should be disclosed, eg, RNA Integrity Numbers (RIN) for any RNA that was obtained, and detailed protocols of downstream methods need to be published, for example, antibody lot numbers in the case of immunostaining. It is also vital to be clear about how samples were batched—this can be particularly important in the case of RNA sequencing, where technical batch effects are a nontrivial issue that cannot be overcome with computational tools alone.^12,36^ Finally, authors should be transparent about whether analyses proceeded in a blinded fashion, and if so, at what stage blinding was introduced and how it was maintained.

For all types of data (whether clinical or tissue-derived), we strongly recommend providing the numerical data required for future systematic reviews and meta-analyses. This includes, but is not limited to, a clear indication of n numbers at participant level (not just number of cells or sections), individual participant data if possible, effect sizes (eg, Cohen *d*, correlations, relative risk), relevant mean values/medians/correlations and corresponding measures of variance (eg, SD or 95% confidence intervals).

Regarding data sharing, it was clear that our attendees routinely practised within-consortium data sharing with the aid of legal and regulatory agreements across individual project partners. REDCap^45^ was the preferred choice of database for these endeavours. This is because it is internationally available, relatively cheap and standardised, with common questionnaires already available, and the possibility of uploading tissue-specific data. The challenges of clinical data sharing with the wider research-community were extensively discussed. They include differences in legal and ethical approval processes for data sharing across countries, potential use of data for nefarious purposes like papermills, and the risk of de-anonymisation. Genetic data pose a special challenge in this regard, particularly when it comes to rarer neuropathic pain conditions including syndromes like erythromelalgia. Positive examples of how wider clinical data sharing can work with appropriate ethical safeguards in place are the Genomics England research environment, UK Biobank, SPARC (an NIH-funded data portal), and the Alleviate Datahub (connecting UK pain-relevant data). All of them have strict rules about who gets access and how—data are often accessible within trusted research environments only and cannot be downloaded. Ultimate say over the data usually remains with the original data controller. It was agreed that such systems are extremely useful, but that they are expensive to set up and maintain. To ensure interoperability, they also need to conform to standardized data models, like OMOP^42^ and CDISC.^7^ They therefore require long-term buy-in from governments and other funding organisations.

Beyond clinical data, sharing of results derived from tissue is often more straightforward. Sequencing data are often the easiest, with data sharing of transcriptomic and proteomic data the rule rather than the exception. A wealth of databases are already available, like Gene Expression Omnibus,^22^ the European Nucleotide Archive,^19^ the Broad Institute Single Cell Portal,^59^ and dbGAP.^13^ It was noted that the last can be difficult to access from outside the United States. It was also suggested that in cases of sensitive sequencing data that is at risk of de-anonymisation, processed data can be made safely available, with fastq files held back in certified cold storage. Flow cytometry data should also be relatively straightforward to share, as common file types like .fcs are not too large. By contrast, raw data sharing of immunostaining images was considered a much greater challenge because of image size and the associated storage costs. However, event attendees agreed that in the age of artificial intelligence, it might be desirable to start sharing images because they could serve to form the large training datasets that will likely be required for automated image analysis and annotation. Similarly, it would be desirable to share primary electrophysiological data to facilitate meta-analysis, with current reviews of the field limited to systematic review methods^8,31^ because of the unavailability of raw results. Finally, there was clear consensus that data sharing without adequate meta-data was pointless. Groups therefore must make an effort to annotate, eg, their individual .fcs files and images, at the very least with a few parameters in an associated meta-data text or Excel file.

## 6. Conclusion

In this white paper, we reported on a consensus process undertaken by a network of neuropathic pain experts, including basic and clinical scientists, as well as patient and industry partners.

We successfully incorporated existing guidelines and reached consensus on the phenotyping guidelines displayed in Table 1. We also decided to share many of our current protocols in future-proof fashion, ie, as version-controlled, living documents.

We hope that our deliberations and recommendations will help integrate human bio-sample research in neuropathic pain. Our ambition is for the field to work together, using harmonized protocols within and across collaborative research efforts. Only collectively will we be able to fully understand the mechanisms that drive neuropathic pain, improve biomarkers of neural injury and consequent neuropathic pain, and, crucially, identify better treatment approaches.

## Conflict of interest statement

R.B. has received Grants/Research support from EU Projects: „Europain” (115007). DOLORisk (633491). IMI Paincare (777500). German Federal Ministry of Education and Research (BMBF): Verbundprojekt: Frühdetektion von Schmerzchronifizierung (NoChro) (13 GW0338C). German Research Network on Neuropathic Pain (01EM0903). Pfizer Pharma GmbH, Grünenthal GmbH, Mundipharma Research GmbH und Co. KG., Alnylam Pharmaceuticals, Inc, Zambon GmbH, Sanofi Aventis GmbH. R.B. has received speaker fees from Pfizer Pharma GmbH, Sanofi Aventis GmbH, Grünenthal GmbH, Mundipharma, Lilly GmbH, Desitin Arzneimittel GmbH, Teva GmbH, Bayer AG, MSD GmbH, Seqirus Australia Pty. Ltd, Novartis Pharma GmbH, TAD Pharma GmbH, Grünenthal SA Portugal, Grünen¬thal Pharma AG Schweiz, Grünenthal B.V. Niederlande, Evapharma, Takeda Pharmaceuticals International AG Schweiz, Ology Medical Education Netherlands, Ever Pharma GmbH, Amicus Therapeutics GmbH, Novo Nordisk Pharma GmbH, Chiesi GmbH, Stada Mena DWC LLC Dubai, Hexal AG, Viatris, AstraZeneca GmbH, Sandoz. R.B. has received consultation fees from Pfizer Pharma GmbH, Sanofi Aventis GmbH, Grünenthal GmbH, Lilly, Novartis Pharma GmbH, Bristol-Myers Squibb, Biogenidec, AstraZeneca GmbH, Daiichi Sankyo, Glenmark Pharmaceuticals S.A., Seqirus Australia Pty. Ltd, Teva Pharmaceuticals Europe Niederlande, Teva GmbH, Genentech, Mundipharma International Ltd. UK, Galapagos NV, Kyowa Kirin GmbH, Vertex Pharmaceuticals, Inc, Biotest AG, Celgene GmbH, Desitin Arzneimittel GmbH, Regeneron Pharmaceuticals Inc. USA, Theranexus DSV CEA Frankreich, Abbott Products Operations AG Schweiz, Bayer AG, Grünenthal Pharma AG Schweiz, Akcea Therapeutics Germany GmbH, Asahi Kasei Pharma Corporation, AbbVie Deutschland GmbH & Co. KG, Air Liquide Sante International Frankreich, Alnylam Germany GmbH, Lateral Pharma Pty Ltd, Hexal AG, Angelini, Janssen, SIMR Biotech Pty Ltd Australien, Confo Therapeutics N. V. Belgium, Merz Pharmaceuticals GmbH, Neumentum, Inc, F. Hoffmann-La Roche Ltd. Switzerland, AlgoTherapeutix SAS France, Nanobiotix SA France, AmacaThera, Inc. Canada, Heat2Move, Resano GmbH, Esteve Pharmaceuticals SA, Aristo. N.A. has received honoraria from Grunenthal, Medtronic, Merz, Novartis outside the submitted work. N.B.F. has received consultancy fees from PharmNovo, Vertex, NeuroPN, Saniona, Nanobiotix, and Neurvati and has undertaken consultancy work for Aarhus University with remunerated work for Biogen, Merz, and Confo Therapeutics. She has received grants from IMI2PainCare an EU IMI 2 (Innovative medicines initiative) public–private consortium and the companies involved are: Grunenthal, Bayer, Eli Lilly, Esteve, and Teva, outside the submitted work. J.R.F.H. is an employee of and owns shares in GSK. P.K. has received honoria from Grunenthal and Vertex Pharmaceuticals and received industry grants from Merck and Lundbeck A/S, outside the submitted work. T.J.P. is a co-founder and holds equity in 4E Therapeutics, PARMedics, Doloromics, Nerveli and NuvoNuro and has received research grants from Grunenthal, Eli Lilly, Merck, Hoba Therapeutics, Bellus Health and Abbvie. C.S. has received honoraria for consulting Algiax, Bayer, Merz, and Omega on the subject of neuropathic pain. RDT reports personal fees from Sanofi, GSK, Grünenthal, CERED, Merz, Bayer, Saluda Medica, Vertex, grants from DFG, EU, TEVA, Esteve as part of EU grant IMI-painCare. E.K.A. is consulting for Genedit and Concentric analgesics and has lectured for Grunenthal. A.L. receives counseling fees from Grünenthal and had a research contract with Grünenthal. D.L.B. has acted as a consultant in the last 2 years for 5 am ventures, AditumBio, Astra Zeneca, Biogen, Biointervene, Combigene, LatigoBio, GSK, Lexicon therapeutics, Neuvati, Novo Ventures, Olipass, Orion, Replay, SC Health Managers, Third Rock ventures, Vida Ventures, Vertex on behalf of Oxford University Innovation. He has received research funding from Lilly and Astra Zeneca. F.D. has consulted for GSK and Cellectricon and received money from Ono Pharmaceuticals. The remaining authors have no conflicts of interest to declare.

## Appendix A. Supplemental digital content

Supplemental digital content associated with this article can be found online at http://links.lww.com/PAIN/C157.
