# Supplementary material for: Harmonizing neuropathic pain research: outcomes of the London consensus meeting on peripheral tissue studies
Source: Pain. 2024 Oct 16;166(5):994–1001. doi: 10.1097/j.pain.0000000000003445 (PMC12004985; doi:10.1097/j.pain.0000000000003445)
Supplement: Supplementary file 1 [file jop-166-0994-s001.pdf]

# ERA-NET Neuron - EndPain Survey results

- Sent out to 33 network partners in June 2023
- 19 responses, 57%

# Do you have access to human tissue/samples?

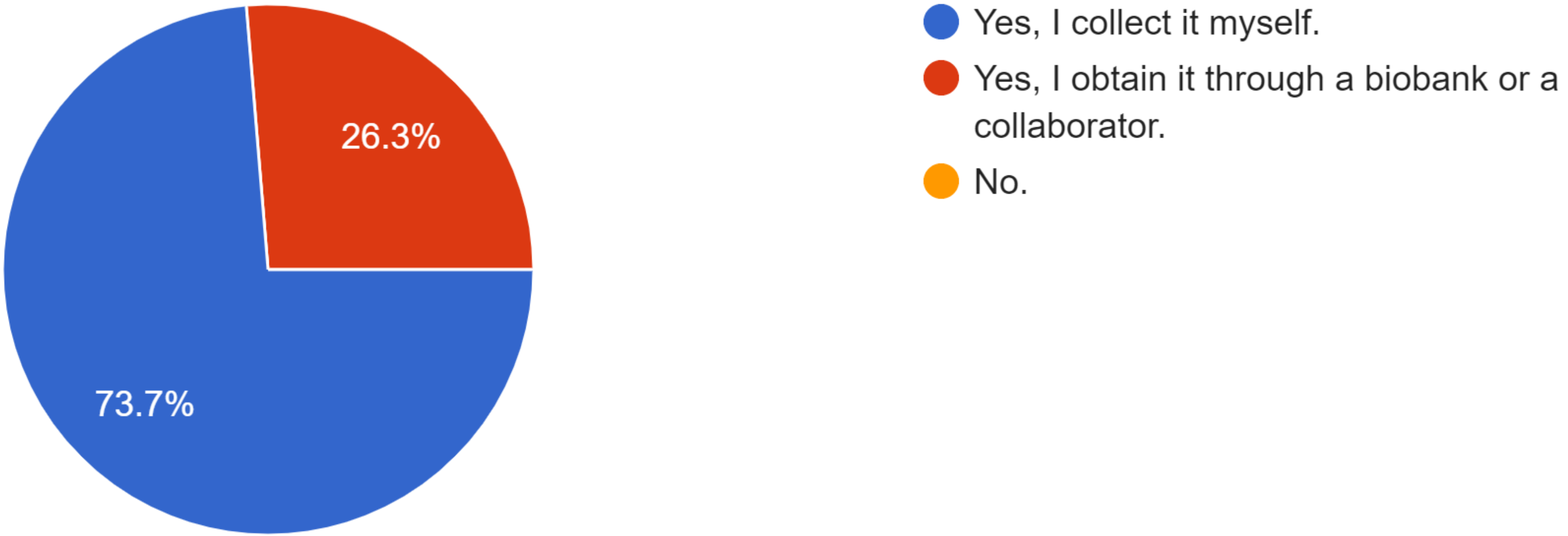

# What kind of tissue do you currently have access to?

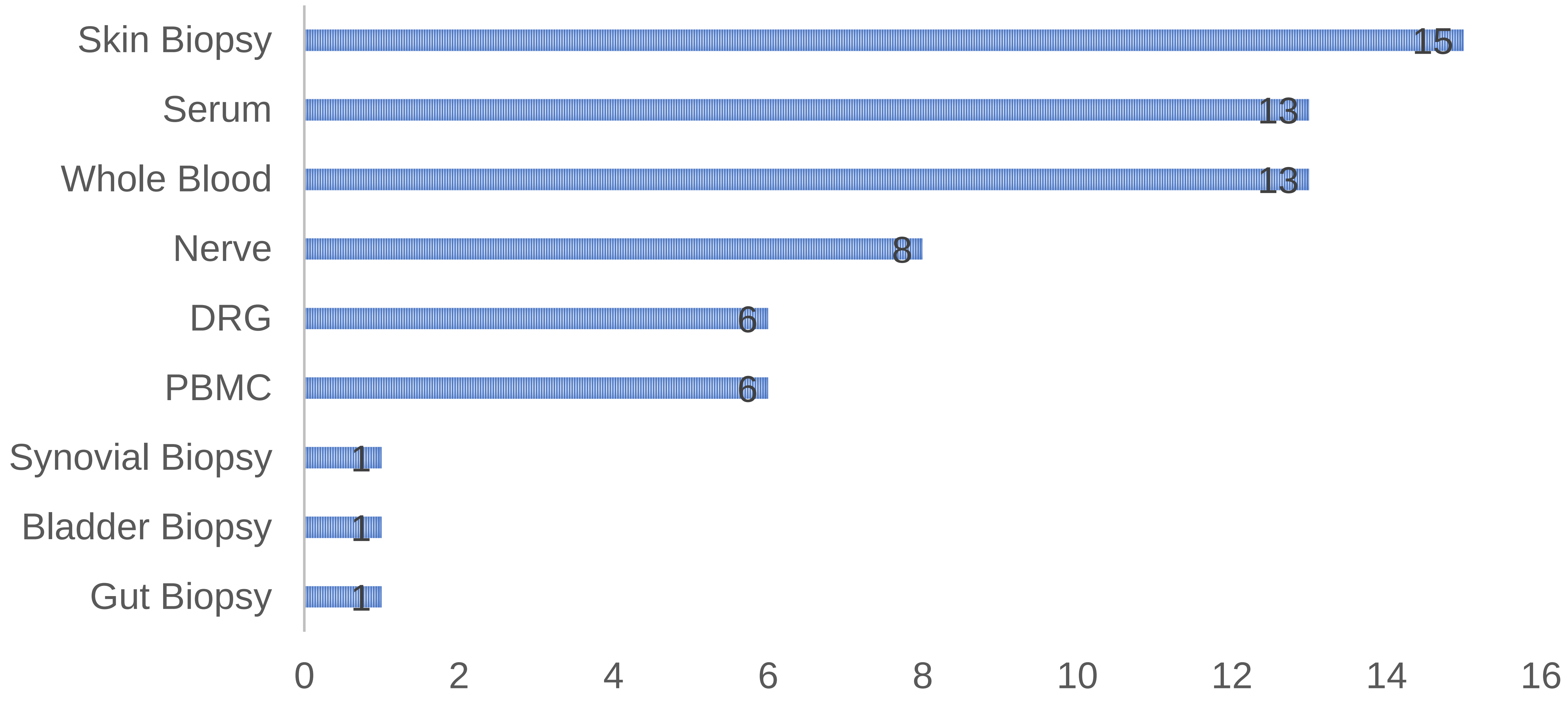

# What kind of tissue do you currently have access to?

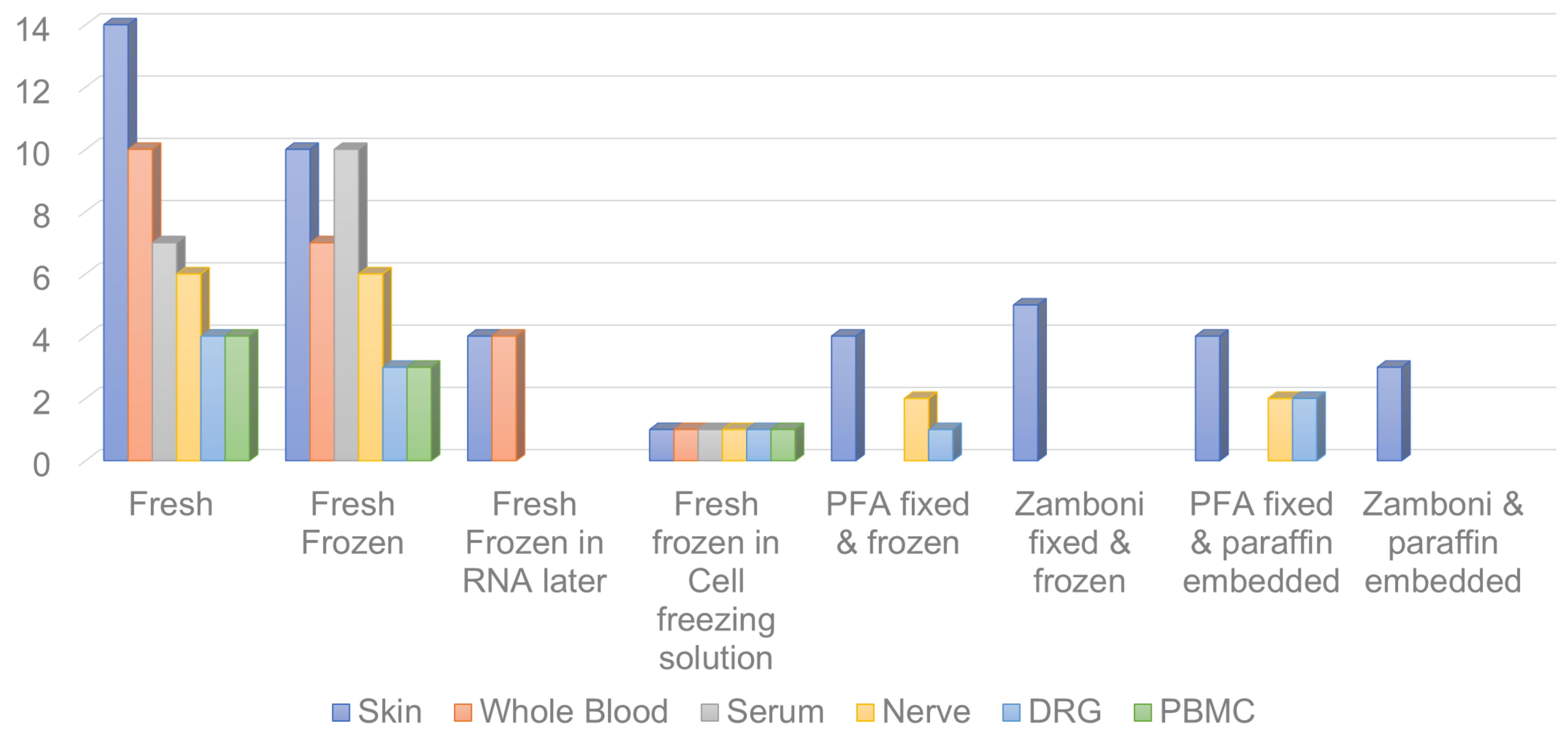

# Which types of samples do you MOST OFTEN collect (top 2)?

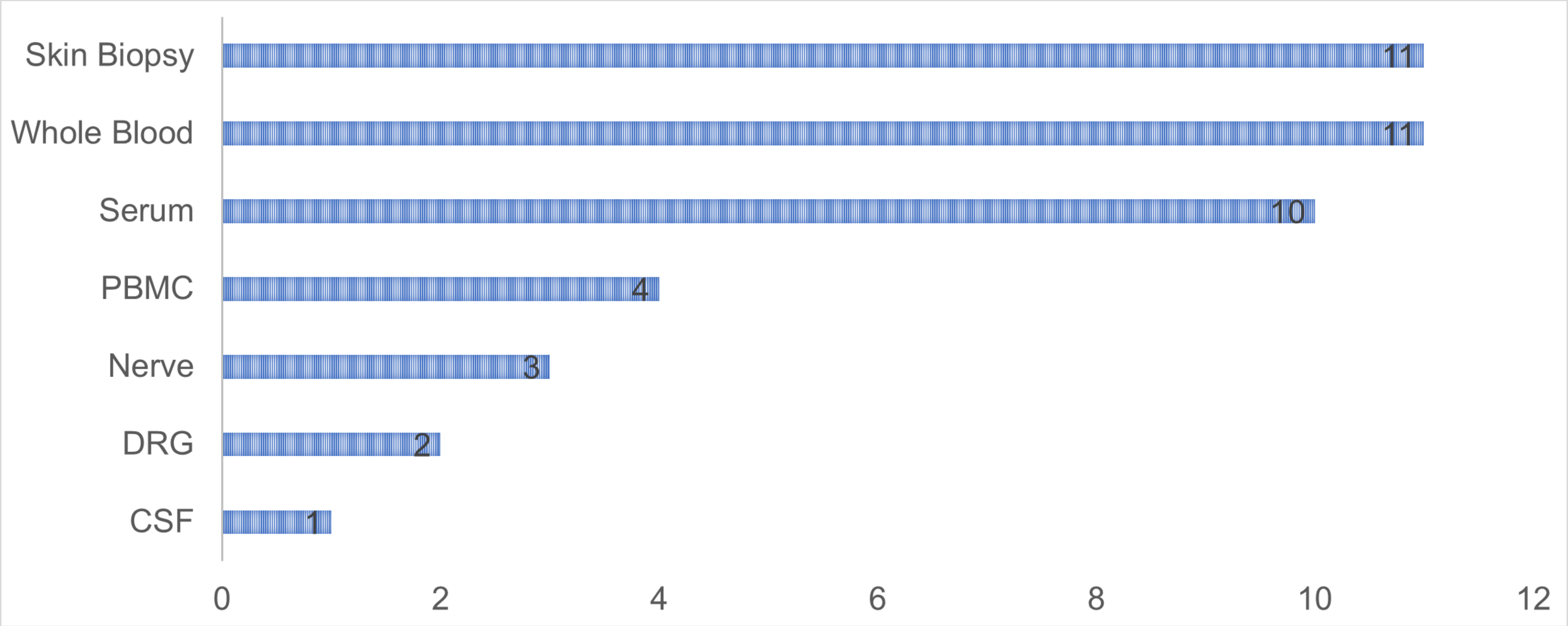

# Do you collect samples longitudinally?

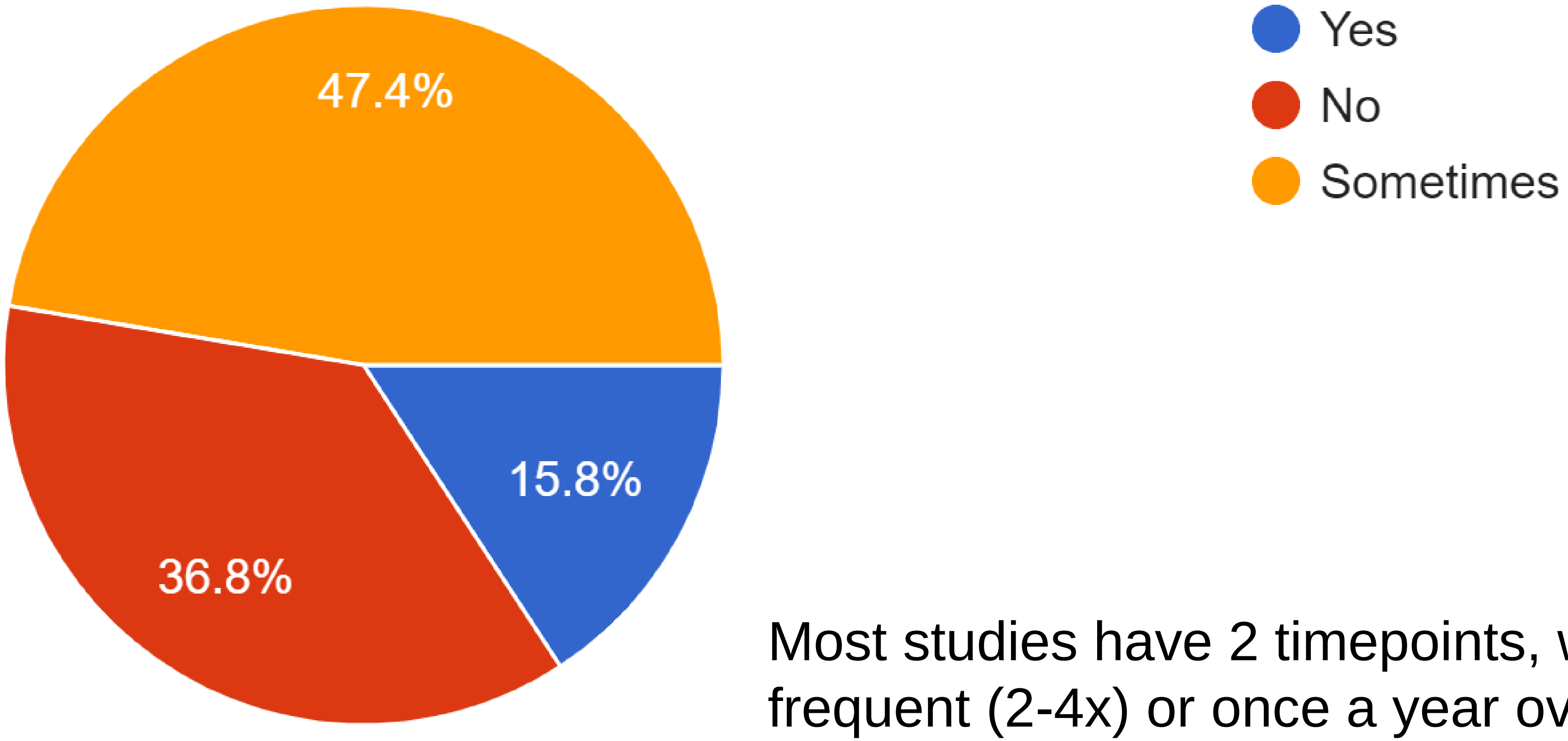


Most studies have 2 timepoints, with a few more frequent (2-4x) or once a year over three years.

# Which types of post-mortem samples do you MOST OFTEN use?

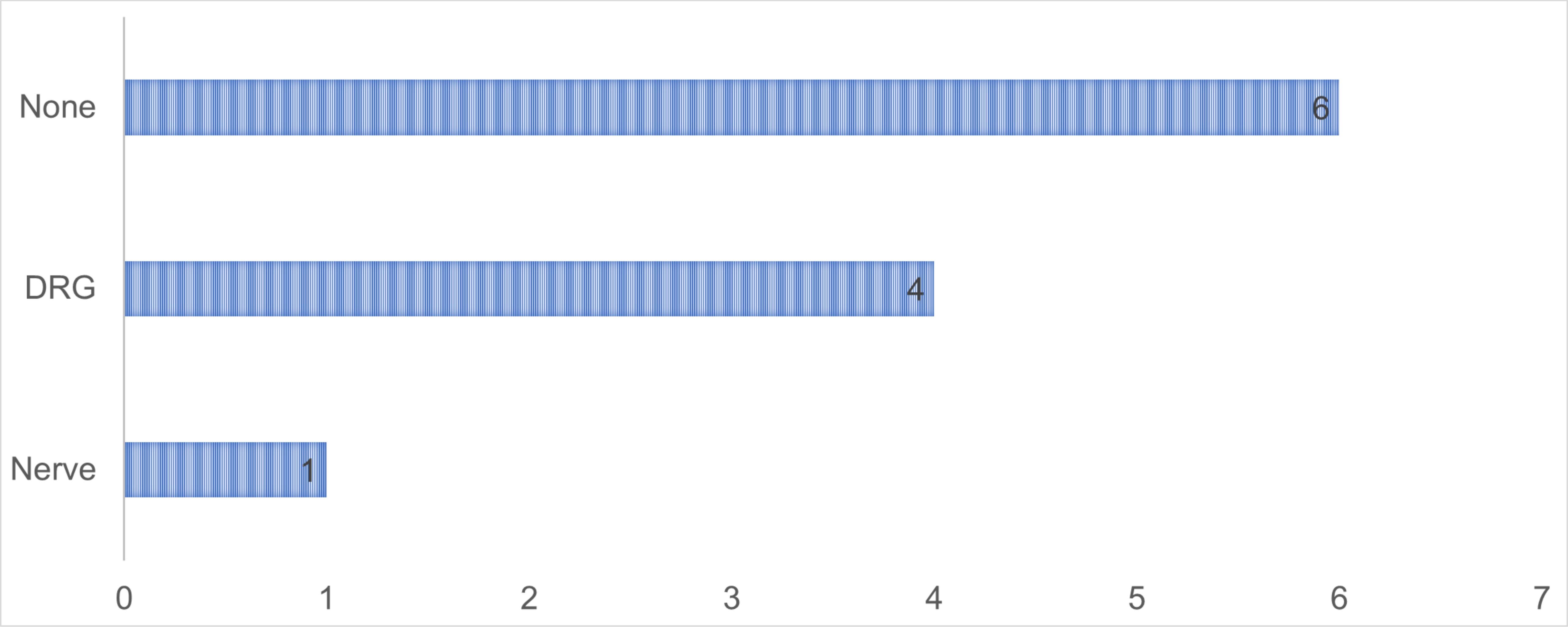

# Types of phenotyping data available with postmortem samples

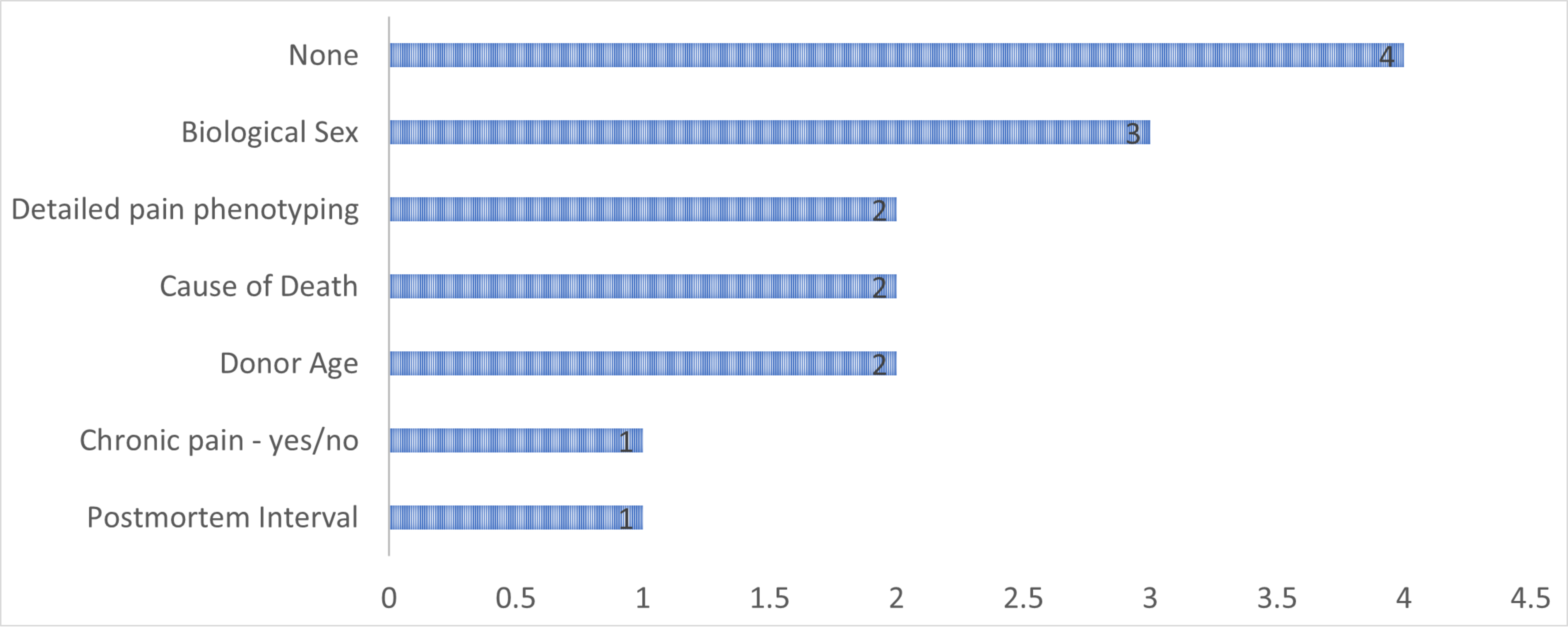

# Cell types currently being studied by respondents in the available tissues/samples

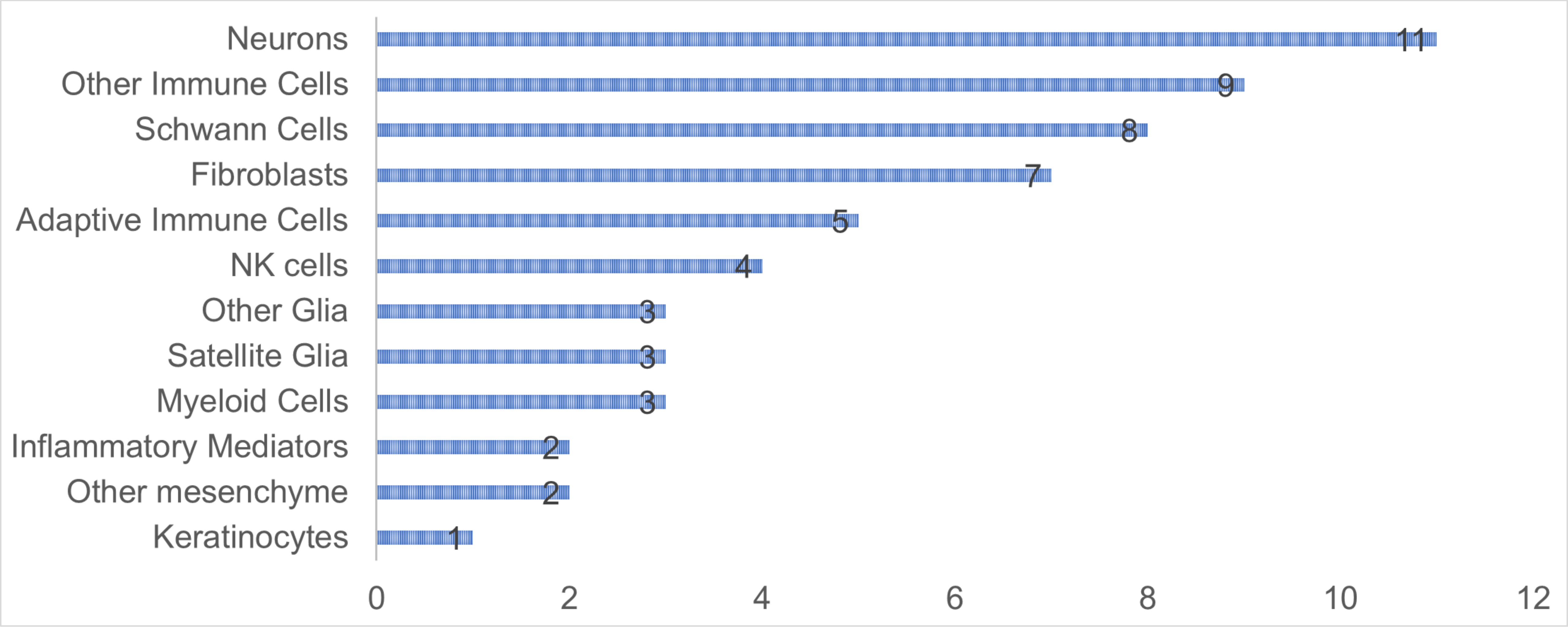

# Phenotyping data being collected

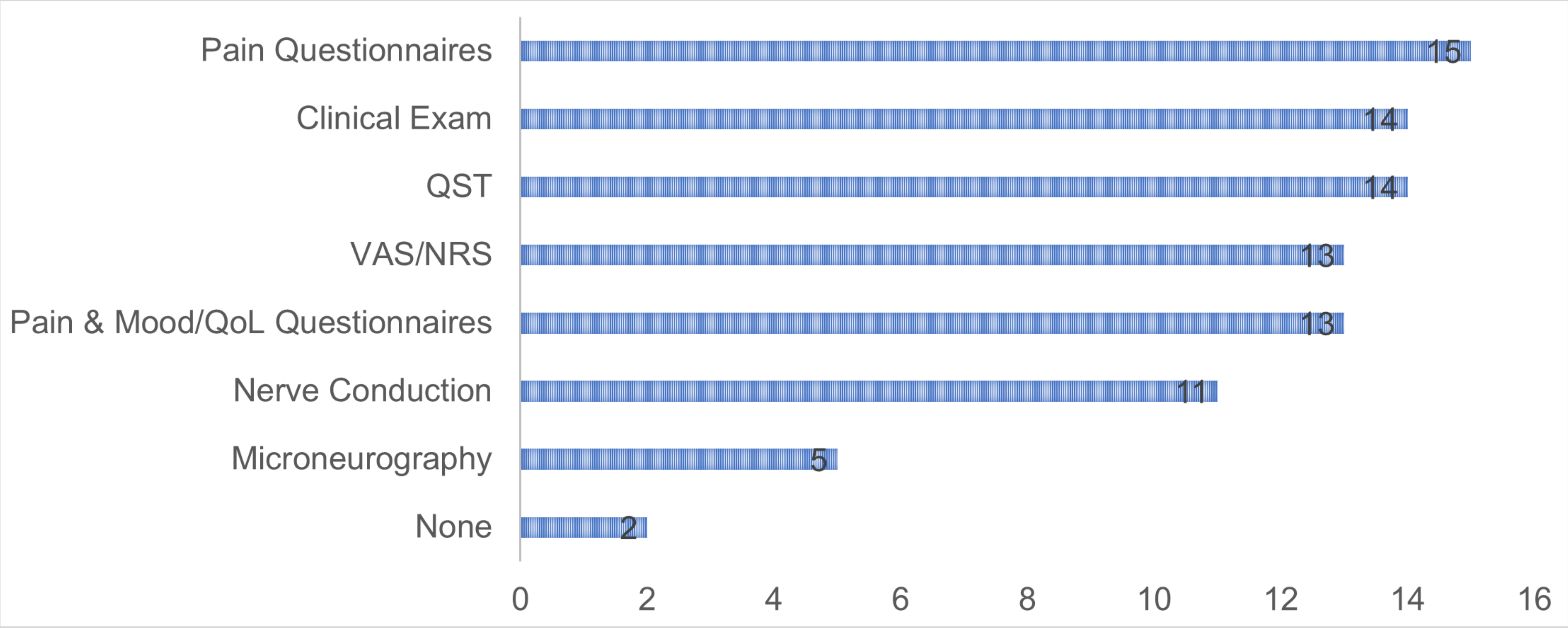

# Favourite Questionnaires

| | |
|---|---|
| painDetect | 4 |
| DN4 | 4 |
| NPSI or NPS | 4 |
| BPI | 3 |
| PROMIS | 3 |
| HADS | 2 |

# If QST, which tests?

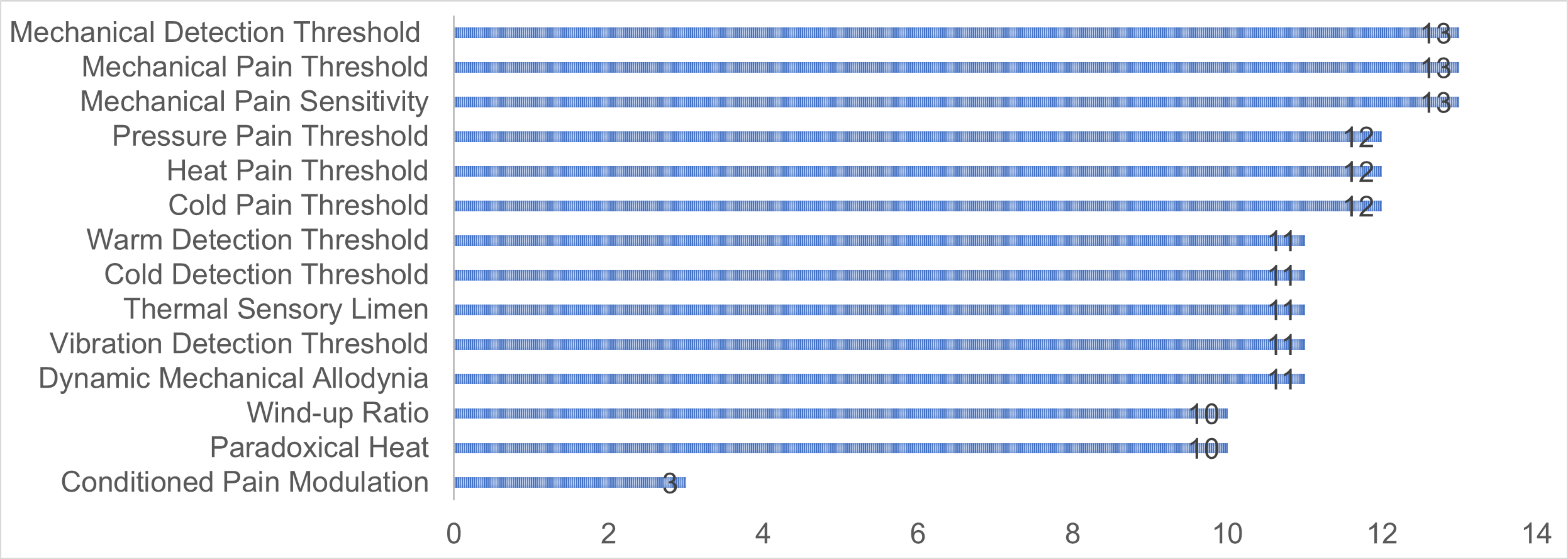

# Does your current ethic/regulatory framework make it hard to share?

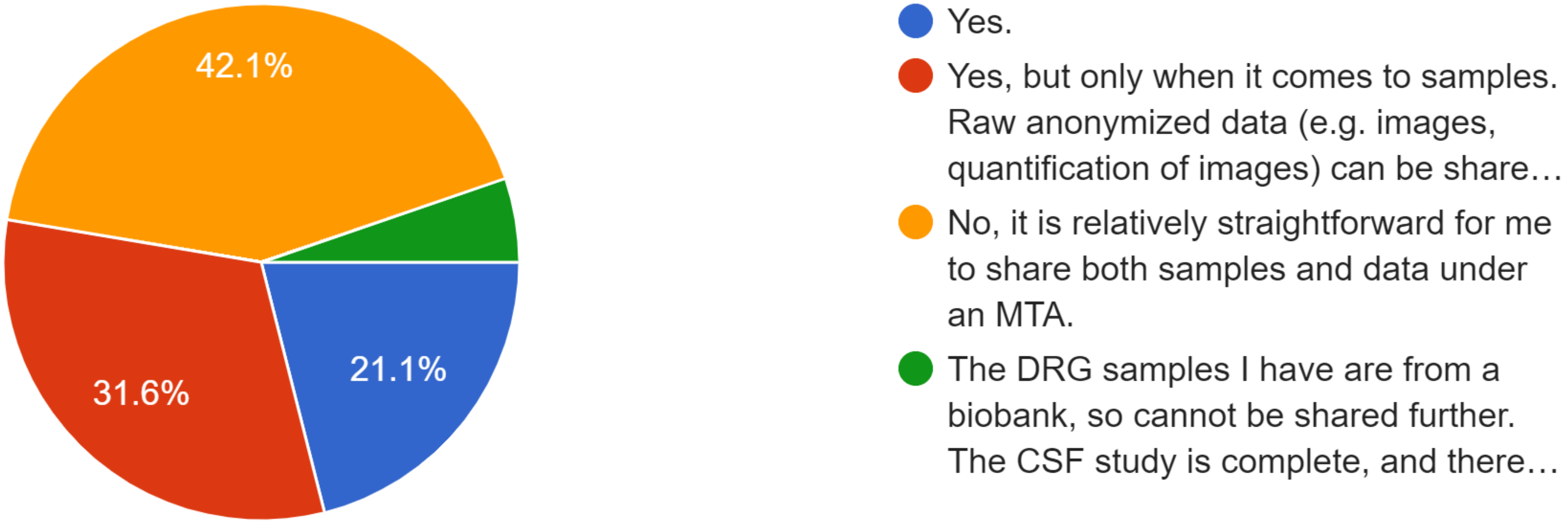

# Human peripheral tissues in pain research
# - Which cell types should the field focus on (top 3)?

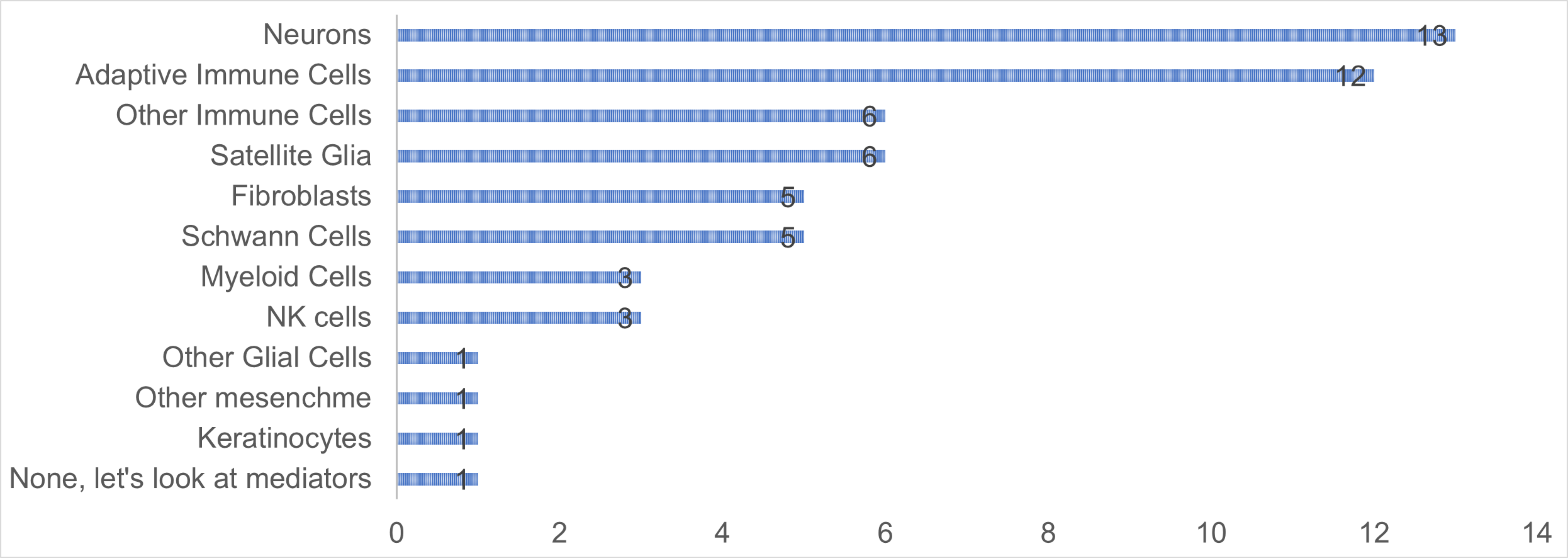

# Top three scientific questions – most common categories

- **Focus on C fibres & ectopic activity, e.g.**
  - Relate C fibre neurophysiology to clinical phenotype;
  - Subtypes of human nociceptors and how they change in neuropathic pain.
  - How and where is the ap generated in the peripheral nerve fiber, and how is spontaneous activity generated?
  - How do changes in the cellular composition of the DRG correlate to pain?

- **Focus on inflammation/ non-neuronal cells in periphery, e.g.**
  - Why does nerve injury and inflammation not always induce pain?
  - How adaptive immune cells communicate to nociceptors?
  - Put together an atlas of human immune cell involvement in health and disease
  - The role of the immune system in the early stages/initiation of peripheral nerve injury that is associated with PAIN not solely NEUROPATHY
  - How do fibroblasts interact with neurons and immune cells to modulate chronic pain?
  - How do sensory neurons cross talk with Schwann cells?

  **& how nociceptors talk back:**
  - Defining the factors that nociceptors release when they fire action potentials.

# Top three scientific questions – most common categories

- **Focus on biomarkers & risk factors**
  - Phenotyping pain patients, stem cells to individualize treatment, biomarker as endpoints
  - What phenotypes predict treatment efficacy?
  - Why some people with neuropathy have pain and others don´t.
  - Personalised Medicine & Risk Factors
  - Are there cellular markers which can predict chronic pain?

- **Focus on models & technology improvement**
  - How can we gain access and process to cadaveric material and link to phenotypic information?
  - Improvement in rigour
  - Species differences (models vs chronic disease in human)
  - What specific cell types do we generate from ipsc when differentiating?

# Biggest obstacles to data/study harmonization (top 2)?

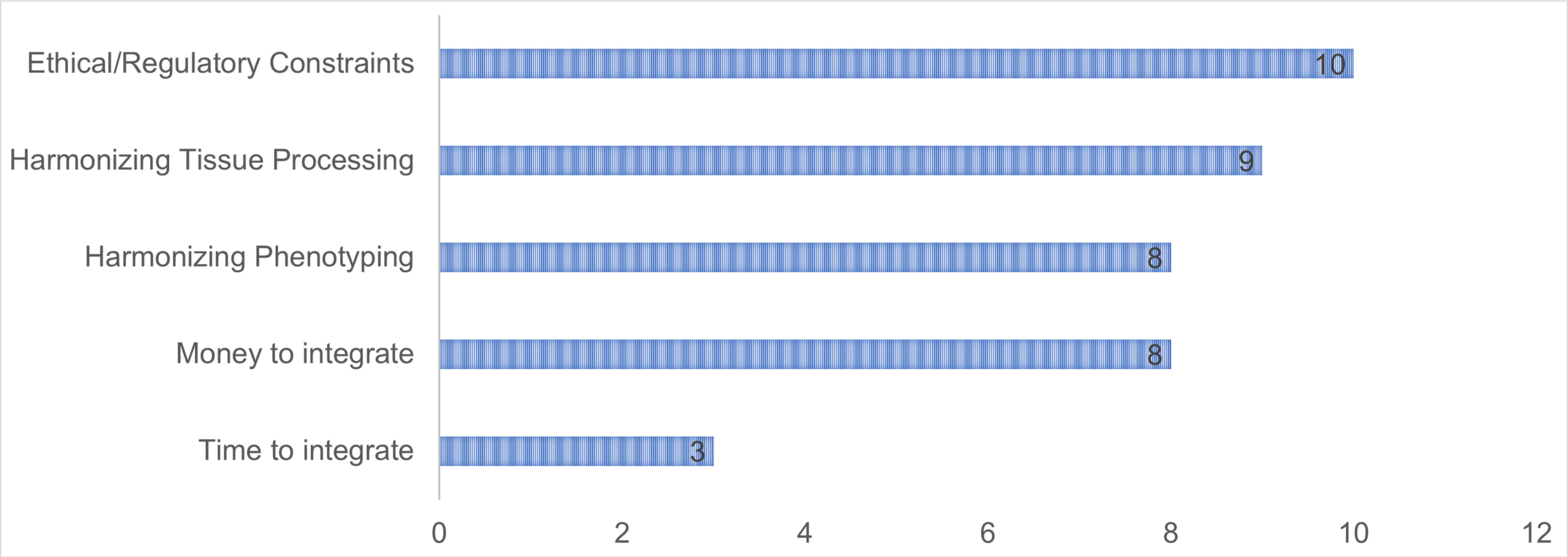

# Willingness to set aside tissue for joint harmonized experiment?

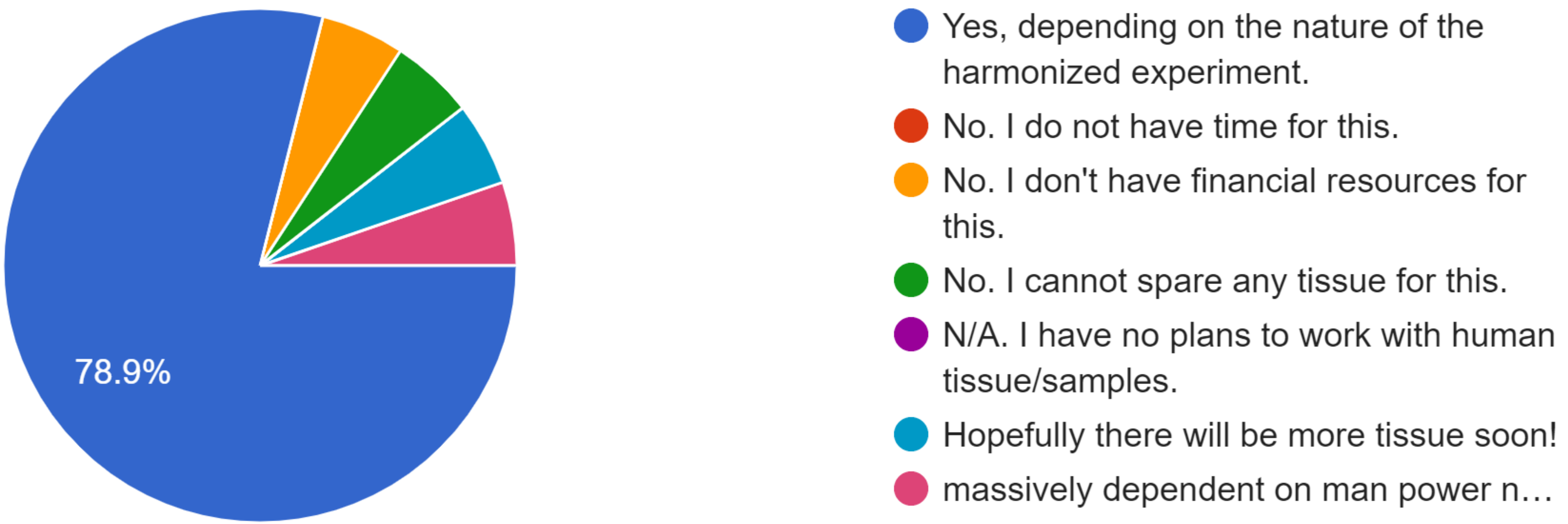

# Dream harmonized experiment?

| | |
|---|---|
| 8x | harmonise work with skin biopsies beyond neurons to expand into immune cells/fibroblasts |
| 1x | clinical phenotype to match receptor profiling |
| 1x | scRNAseq of human DRG across ages/diseases |
| 3x | focus on harmonize methods instead (i.e. biobank) |
| 6x | no answer |

**Minimal Pain Phenotyping required?**
Most answers not particularly focused on minimal…mention of questionnaires, alongside clinical/demographic information as well as QST.

Good summary of pain phenotyping perhaps this example:
*‘Pain type (e.g. neuropathic pain grading), pain intensity, pain descriptors, possible sensory testing (including suprathreshold)’*

# How to tackle batch effects?

| | |
|---|---|
| Common Protocols | 7 |
| Sentinels | 3 |
| Common Markers/Antibodies | 5 |
| Large & Diverse Sample | 1 |
| Too challenging | 1 |

- Beyond the above, would it help if focus was on cell counts?
- We could generate reference values akin to IENF reference values?

# Is a biobank feasible?

- The group largely agrees it would be valuable (15/18 answers, only 1 no)
- Clear caveat that it would be very hard to set up/finance (5/18 answers)
- Everyone wants standardised phenotyping data in that case (11/18 answers)

# Human sample collection form

| | |
|---|---|
| Date: | |
| Research site (incl. institution, city, country): | |
| | *Circle as applicable:* |
| Confirm consent signed: | Yes No |
| Consent to share samples: | Yes No |
| Type of sample: | Blood Skin Nerve |

***For skin and nerve:*** *Circle as applicable:*

| | |
|---|---|
| Which side: | Left Right |
| Body site and location (for skin biopsy): *e.g. 10cm proximal to lateral malleolus of the leg/finger* | |
| Biopsy taken at site of neuropathic pain? | Yes No |
| Which nerve (for nerve biopsy): | Sural Superficial Peroneal Superficial Radial<br>Other (please specify): |
| Samples in | PLP flash frozen RNA later<br>Other (please specify): |

***For blood:***

| | |
|---|---|
| Whole blood (EDTA): | ml |
| Serum: | ml |
| Plasma: | ml |
| RNA: Paxgene/Tempus tubes | ml |
| Time of last meal: | min before blood taken |

***Essential clinical information:***

| | |
|---|---|
| Diagnosis: | |
| Age | |
| Biological sex | |
| Height/weight | cm kg |
| Specify main symptoms, including symptom location on body map: | 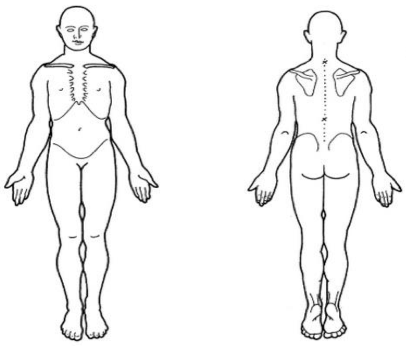 |

| | |
|---|---|
| Pain severity at site of neuropathic pain (0-10) | |
| Duration of symptoms (in months) | |
| **Medication history** (past or present neurotoxic medications and/or treatments, current analgesics and strong immunomodulators) | |
| **Neuropathic pain grading** | Unlikely Possible Probable Definite |
| **Results of clinical examination (e.g., neurological screen)** | |

***Any other relevant information*** *(example provided for diabetic neuropathy)****:***

| | |
|---|---|
| Diabetes: | Yes No |
| HbA1c (mmol/mol and %) for diabetes (including date): | |
| Clinical neurophysiology results summary: | |

| | |
|---|---|
| **Researchers name:** | **Contact email address:** |
| | |
