# Supplementary material for: Harmonizing neuropathic pain research: outcomes of the London consensus meeting on peripheral tissue studies
Source: Pain. 2024 Oct 16;166(5):994–1001. doi: 10.1097/j.pain.0000000000003445 (PMC12004985; doi:10.1097/j.pain.0000000000003445)
Supplement: Supplementary file 2 [file jop-166-0994-s002.pdf]

Open Access License Agreement

This OPEN ACCESS LICENSE AGREEMENT (this "Agreement"), dated as of.

12/09/2024

DATE

(the "Effective Date"), by and between Wolters Kluwer Health, Inc., operating as Medical Research / Lippincott Williams & Wilkins, a Delaware corporation, having its principal place of business at Two Commerce Square, 2001 Market Street, Philadelphia, PA 19103 (the "Publisher"), and the corresponding author listed on Schedule A to this Agreement (the "Author", and together with the Publisher, the "Parties").

1. Grant of License

The Author hereby grants to the Publisher and its Affiliates the exclusive, worldwide, royalty free, perpetual (for the duration of the applicable copyright) right and license to use the Work for all commercial or educational purposes, including, but not limited to, publishing, reproducing, marketing, distributing (themselves and through distributors), sublicensing, and selling copies of the Work throughout the world for the Term. If the Author is a United States government employee, such license grant shall be limited to the extent the Author is able to grant such license.

2. Warranties, Indemnification, and Limitation of Liability

a. The Author represents and warrants that:

(i) it has the right and power to enter into this Agreement, to grant the rights and licenses granted pursuant to this Agreement, and to perform all of its other obligations contained in this Agreement;

(ii) it has not previously assigned, transferred or otherwise encumbered the rights or licenses granted pursuant to this Agreement; and that the person executing this Agreement on the Author's behalf is authorized to do so;

(iii) the Work and the licenses granted herein do not and will not infringe upon, violate or misappropriate any intellectual property rights or any other proprietary right, contract or other right or interest of any third party;

(iv) if the Work is a multi-authored Work, the Author has obtained written permission from each author of the Work to enter into this Agreement on behalf such author, and each such author has read, understands and has agreed to the terms of this Agreement; and

(v) the Author has obtained any necessary releases and permissions to quote from other sources in the Work and to include any works and materials in the Work and all such releases and permissions are in full force and effect.

b. The Author hereby indemnifies the Publisher and its directors, officers, employees, agents, and representatives and agrees to defend and hold them harmless from and against any and all liability, damage, loss, costs or expenses (including reasonable attorney's fees and costs of settlement) incurred by any such party arising out of, or relating to any misrepresentation in, or breach or alleged breach of the Author's representations or warranties in this Agreement. If the Author fails to promptly or diligently pursue any defense of any indemnified party, the indemnified parties, or any of them, may assume such defense at the Author's expense. The obligations of this indemnification will survive any termination or expiration of this Agreement.

c. The Publisher represents and warrants that it has the right and power to enter into this Agreement and to perform its obligations contained in this Agreement, and that the person executing this Agreement on the Publisher's behalf is authorized to do so.

d. The Publisher hereby indemnifies the Author and agrees to defend and hold the Author harmless from and against any and all liability, damage, loss, costs or expenses (including reasonable attorney's fees and costs of settlement) incurred by the Author arising out of, or relating to any misrepresentation in, or breach or alleged breach of the Publisher's representations or warranties in this Agreement. If the Publisher fails to promptly or diligently pursue any defense of the Author, the Author may assume such defense at the Publisher's expense. The obligations of this indemnification will survive any termination or expiration of this Agreement.

e. EXCEPT AS OTHERWISE SET FORTH IN THIS AGREEMENT, NEITHER PARTY MAKES ANY OTHER, AND HEREBY DISCLAIMS ALL OTHER, REPRESENTATIONS AND WARRANTIES OF ANY KIND, WHETHER EXPRESS, IMPLIED, STATUTORY OR OTHERWISE, INCLUDING, WITHOUT LIMITATION, WARRANTIES OF TITLE, MERCHANTABILITY, FITNESS FOR A PARTICULAR PURPOSE, NONINFRINGEMENT, OR THE ABSENCE OF LATENT OR OTHER DEFECTS, ACCURACY, OR THE PRESENCE OR ABSENCE OF ERRORS, WHETHER OR NOT DISCOVERABLE.

f. EXCEPT TO THE EXTENT REQUIRED BY APPLICABLE LAW, IN NO EVENT WILL EITHER PARTY BE LIABLE TO THE OTHER PARTY BASED UPON ANY LEGAL THEORY FOR ANY SPECIAL, INCIDENTAL, CONSEQUENTIAL, PUNITIVE, OR EXEMPLARY DAMAGES ARISING OUT OF THIS LICENSE OR THE USE OF THE WORK, EVEN IF A PARTY HAS BEEN ADVISED OF THE POSSIBILITY OF SUCH DAMAGES.

3. Creative Commons License.

Creative Commons Licenses are subject to items selected in item 1, 2 and 3 in the Schedule B.

a. CCBY-NC-ND – NonCommercial-NonDerivitives Creative Commons License

The Author acknowledges and agrees that the Work will be published by the Publisher in (the "Journal") and made freely available to users under the terms of the Attribution-NonCommercial-NoDerivs 4.0 Creative Commons License, as currently displayed at http://creativecommons.org/licenses/by-nc-nd/4.0/legalcode (the "CC BY-NC-ND"). The Author acknowledges and agrees that that Publisher is the exclusive "Licensor", as defined in the CC BY-NC-ND, of the Work and that the Publisher may make the Work freely available to all users under the terms of the CC BY-NC-ND.

b. CCBY – Creative Commons License

The Author acknowledges and agrees that the Work will be published by the Publisher in (the "Journal") and made freely available to users under the terms of the Attribution 4.0 Creative Commons License, as currently displayed at http://creativecommons.org/licenses/by/4.0/legalcode (the "CC BY"). The Author acknowledges and agrees that that Publisher is the exclusive "Licensor", as defined in the CC BY, of the Work and that the Publisher may make the Work freely available to all users under the terms of the CC BY.

4. Royalties.

The Author acknowledges and agrees that this Agreement entitles the Author to no royalties or fees. To the maximum extent permitted by law, the Author waives any and all rights the Author may have to collect royalties or other fees in relation to the Work or in respect of any use of the Work by the Publisher or its sublicensees.

5. Miscellaneous.

a. Assignment. This Agreement may not be assigned or transferred, in whole or in part, by either party without the prior written consent of the other party. Notwithstanding the above, the Publisher may assign this Agreement without the written consent of the Author (i) to an entity succeeding, whether by sale, merger or other corporate reorganization, to substantially all of the Publisher's assets and business activity, or (ii) to a corporation or organization that obtains the right to publish the Journal from the Publisher. The Publisher may assign this Agreement to any of its affiliates. This Agreement will be binding upon and inure to the benefit of the parties hereto and their respective successors and permitted assigns.

b. Counterparts. This Agreement may be executed in two or more counterparts, each of which shall be deemed an original, but all of which together shall constitute one and the same document. Facsimile or Portable Document Format (PDF) signatures will be deemed original signatures for purposes of this Agreement.

c. Entire Agreement; Amendment. This Agreement sets forth the entire agreement of the parties on the subject hereof and supersedes all previous or contemporaneous oral or written representations or agreements relating to the rights and duties provided herein, and may not be modified or amended except by written agreement of the parties.

d. Force Majeure. Neither party shall be liable for any default or delay on its part in performing any obligation under this Agreement if such default or delay is caused by natural disaster, accident, war, civil disorder, strike or any other cause beyond the reasonable control of such party. In the event that either party is prevented by such an occurrence or circumstance for a period of more than ninety (90) days from fulfilling its obligations under this Agreement, the other party may terminate this Agreement upon thirty (30) days' written notice.

e. Governing Law. This Agreement shall be governed in all respects according to the laws of the State of New York without giving effect to the principles of conflict of law thereof.

f. Headings. All headings are for reference purposes only and shall not affect the meaning or interpretation of any provision hereof.

g. Severability. If any provision of this Agreement is held to be illegal, invalid, or unenforceable under the present or future laws, then such provision shall be revised by a court of competent jurisdiction to be enforceable if permitted under applicable law, and otherwise shall be fully severable. In any event, this Agreement shall be construed and enforced as if such illegal, invalid, or unenforceable provision had never comprised a part of this Agreement, and the remaining provisions of this Agreement shall remain in full force and effect and shall not be affected by the illegal, invalid, or unenforceable provision or by its severance from this Agreement.

h. Status of the Parties. The parties are independent contractors. Nothing in this Agreement is intended to or shall be construed to constitute or establish any agency, joint venture, partnership or fiduciary relationship between the parties, and neither party has the right or authority to bind the other party nor shall either party be responsible for the acts or omissions of the other.

i. Waiver; Amendment. The waiver by either party of or the failure by either party to claim a breach of any provision of this Agreement shall not be, or be held to be, a waiver of any subsequent breach or affect in any way the further effectiveness of any such provision. No term or condition of this Agreement may be waived except by an agreement by the parties in writing.

j. Waiver of Jury Trial. EACH PARTY HEREBY WAIVES ITS RIGHT TO A JURY TRIAL IN CONNECTION WITH ANY DISPUTE OR LEGAL PROCEEDING ARISING OUT OF THIS AGREEMENT OR THE SUBJECT MATTER HEREOF.

[Signature Page Follows]

Schedule A

This Schedule A must be completed by Author in its entirety. The Publisher is unable to publish the Work unless this Schedule A is completely filled out.

PAIN-D-24-00603R1

Article Tracking #

The Local Molecular Signature of Human Peripheral Neuropathic Pain

Article Title (the "Work")

Annina Schmid

Corresponding Author Name (the "Author")

Annina Schmid

Copyright Owner's Name

Pain

Name of Journal in which Work is to be Published

Schedule B

This Schedule B must be completed by Author in its entirety. The Publisher is unable to publish the Work unless this Schedule B is completely filled out.

MANDATED FUNDING POLICY DISCLOSURE

1. Choose a funder from the drop down list. If your funder is not shown please complete option 2 below. Corresponding authors who have provided an affiliation that matches an institution listed here* should select 'ULA' from the drop down list. *Please note Authors from the listed Swiss institutions may choose either ULA from the drop down or select option 2 below.

Wellcome Trust

NOTE: If you are a World Health Organization Employee and are required to publish under the Creative Commons CCBY IGO license, then do not complete this form. Instead, please contact the Editorial Office for the separate WHO Employee license agreement.

NOTE: If you have selected a funder or 'ULA' from the drop down menu then the Work will be published under the CCBY license i.e., Section 3.b. of the Agreement will apply to the Work, and Section 3.a. of the Agreement will not apply to the Work.

2. ☐ This Schedule B is inapplicable to the Work.

NOTE: If author has selected option 2, the Work will be published under the CCBY-NC-ND license i.e., Section 3.a. on the Agreement will apply to the Work, and Section 3.b. of the Agreement will not apply to the Work.

GOVERNMENT EMPLOYEES

3. ☐ This work was created in the course of an author's employment by the United States Government

If the Work or a portion of it has been created in the course of any author's employment by the United States Government, check the "Government" box at the end of this form. A work prepared by a government employee as part of his or her official duties is called a "work of the U.S. Government" and is not subject to copyright. If it is not prepared as part of the employee's official duties, it may be subject to copyright.

If "Government" is chosen, please do not choose a Creative Commons License. The work will be published with "Written work prepared by employees of the Federal Government as part of their official duties is, under the U.S. Copyright Act, a "work of the United States Government" for which copyright protection under Title 17 of the United States Code is not available. As such, copyright does not extend to the contributions of employees of the Federal Government."

NOTE: If author has selected option 3, Section 3. on the Agreement will not apply to the Work.

SIGNATURE PAGE

The Corresponding Author acknowledges and agrees that the Corresponding Author is entering into, and has executed, the Agreement on behalf of the Corresponding Author and each other author named as contributing to the Article (each such author, an “Author”, and collectively, the “Authors”). The Corresponding Author represents and warrants that the Corresponding Author has obtained permission from each Author to enter into the Agreement on behalf of such Author and the Corresponding Author and each Author has read, understands, and has agreed to the terms of the Agreement, including, without limitation, the terms contained in the Agreement with respect to authorized reuse of the Article.

IN WITNESS WHEREOF, the Author has executed this License, effective as of the Effective Date.

Annina Schmid

PRINT NAME

Annina Schmid Digitally signed by Annina Schmid
Date: 2024.09.12 17:27:07 +01'00'

SIGNATURE

Important Note: Once you electronically sign this form, you will not be able to make any additional changes to it.

To electronically sign this form, click the signature field above and provide the information requested in the dialog boxes.
